# The heart of darkness: growth and form of *Trypanosoma brucei* in the tsetse fly

**DOI:** 10.1016/j.pt.2009.08.001

**Published:** 2009-11

**Authors:** Reuben Sharma, Eva Gluenz, Lori Peacock, Wendy Gibson, Keith Gull, Mark Carrington

**Affiliations:** 1 Department of Biochemistry, University of Cambridge, 80 Tennis Court Road, Cambridge CB2 1GA, UK; 2 Sir William Dunn School of Pathology, University of Oxford, South Parks Road, Oxford OX1 3RE, UK; 3 School of Biological Sciences, University of Bristol, Bristol BS8 1UG, UK; 4 Faculty of Veterinary Medicine, Universiti Putra Malaysia, 43400 UPM, Serdang, Selangor, Malaysia

## Abstract

The first description of African trypanosomes was made over a century ago. The importance of the tsetse in transmission and cyclic development of trypanosomes was discovered soon afterwards, and has been the focus of numerous studies since. However, investigation of trypanosomes in tsetse flies requires high resource investment and unusual patience; hence, many facets of trypanosome biology in the tsetse remain to be characterised despite the long history of research. Here, current knowledge and questions about some of the developmental changes in trypanosomes that occur in tsetse flies are summarised, along with recent technical advances that can now be used to provide some answers.

## Over a century of discovery

The first scientific description of African trypanosomes was at the end of the nineteenth century by Plimmer and Bradford [1]. From the earliest investigations of trypanosomes present in tsetse flies, it was clear that a range of cellular morphologies were present [2]. *Trypanosoma brucei* morphology was first characterised using light microscopy, the only tool available at the time. Different morphologies were defined on the basis of the: (i) dimensions of the cell body (particularly the length-to-width ratio); (ii) relative positions of the flagellum, nucleus and kinetoplast (concatenated mitochondrial DNA) along the anteroposterior (AP) axis of the cell; and (iii) point of emergence of the flagellum from the cell body [3] (see Box 1 for a description of Trypanosomatid cellular morphologies). Bruce showed the link between trypanosomiasis and tsetse in his studies on ‘the fly disease’ in South Africa, but Kleine was the earliest investigator to elucidate that *Trypanosoma brucei* had to complete a set cycle through a tsetse to produce and transmit forms that would infect mammals [4]. Bruce corroborated this finding for *T. brucei gambiense*, and noted that trypanosomes taken up in a bloodmeal reacquired their infectivity to mammals after a discrete period of at least 12 days within the fly, consistent with a developmental cycle [5]. Bruce went on to describe the developmental stages (morphotypes) present in tsetse, and demonstrated that distinct morphotypes were restricted to different tissues [6]. Robertson and Bruce defined the sequentially occurring morphotypes of *T. brucei gambiense* as it matured in the tsetse vector using morphometry (measurement of cellular features) and illustrations [7–10]. Further detail was added to the life cycle over the next 40 years [11,12].

The beautifully drawn diagram of the life cycle by Keith Vickerman that has become iconic, and will be familiar to any student of parasitology, was originally drawn to incorporate observations made using electron microscopy, the next stage of understanding of the life cycle [13,14]. Ultrastructural features of the various developmental stages were described in detail [13–20]. The major findings came from comparison of trypanosomes from mammals and tsetse, and provided evidence of different cell surfaces in the two hosts and ultrastructural detail. For example, the mitochondrion in trypanosomes from tsetse flies was fully elaborated with cristae, whereas it was smaller and lacked cristae in cells from mammals. This observation correlated with the biochemistry: an active mitochondrion in insect forms is necessary for oxidative catabolism, whereas the mitochondrion is reduced in size and complexity in mammalian forms that predominantly use glycolysis [21].

Here, two of the many questions that remain about trypanosome biology in the tsetse are addressed: (i) what are the mechanisms underlying the changes in trypanosome cellular morphology during the life cycle? And (ii) what are the cellular events necessary for genetic exchange? Some of the original observations from 50–100 years ago are cited throughout to illustrate the need for the use of newly available techniques to answer questions first posed a long time ago. Finally, recent technological advances that hold the prospect of providing some answers are described.

We have restricted ourselves to *T. brucei* because this is the species most amenable to experimentation. Other authors have recently considered differentiation between life-cycle stages [22], and the molecular interactions between trypanosomes and tsetse [23]. The tsetse is parasitized by trypanosomes, and the factors that affect the development of the infection [24] and the response of the tsetse to infection have also been reviewed recently [25]. Other trypanosome species behave differently from *T. brucei* in the tsetse fly, with attachment of epimastigotes to the chitinous lining of the proboscis and cibarium in *T. congolense* and *T. vivax* [26], and hindgut development of metacyclics in *T. grayi*, the tsetse-transmitted crocodile trypanosome [27]. There are no differences in the developmental progression of *T. b. brucei* and the human infective subspecies, *T. b. gambiense* and *T. b. rhodesiense*, although different strains vary in their efficiency of transmission [28].

## Structures that underlie cellular morphology

The shape of a trypanosome results from a spiraled corset of interlinked microtubules that underlies the plasma membrane. This microtubule cytoskeleton must be able to accommodate growth and division of the cell [29], and has to be remodelled to accommodate the different cellular morphologies of the various developmental forms. The sub-pellicular microtubules are oriented with the plus ends at the posterior pole [29]; the exception is the microtubule quartet adjacent to the flagellar attachment zone that originates near the basal body and have plus ends extending to the anterior pole. The cells have a single flagellum that originates at a basal body which is physically connected to the kinetoplast [30]. The flagellum exits the cell through a hole in the microtubule corset. The replication of the flagellum is an integral part of the cell cycle and the separation of the old and new flagellum before cytokinesis requires that the hole(s) through which the two flagella emerge can move relative to one another in the context of the corset. As the new flagellum emerges and then extends along the cell body, the tip remains adjacent to the old flagellum. In procyclic forms, the tip is held on the old flagellum by a structure, the flagella connector [31,32] that, in turn, marks and possibly determines the location where the cytokinetic furrow starts [33].

## Travels in a tsetse and changes in morphology

**Figure 1:**
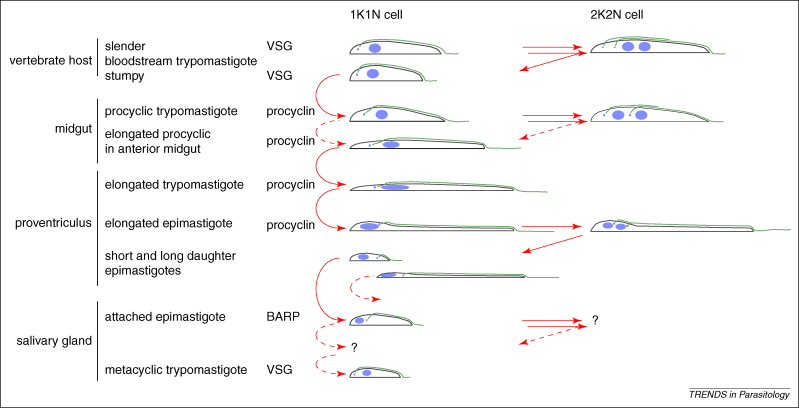
A simplified summary of the morphotypes present in a tsetse, and a comparison with a dividing population in the bloodstream of a mammalian host. Throughout the life cycle, there are a series of transitions between different morphotypes that include a remodelling of cell shape (length and diameter), and a re-ordering of internal organelles. Solid lines are used to indicate transitions that are fairly well characterised; dashed lines indicate alternative or uncharacterised steps. Question marks indicate morphologies that are not fully characterised. Two of the transitions that occur in tsetse flies have been studied in detail, the differentiation of bloodstream to procyclics and, in lesser detail, the asymmetric division that gives rise to epimastigotes. There are four stages shown that undergo cell division: the midgut procyclic forms, salivary gland attached epimastigotes and the mammalian bloodstream forms can all undergo repeated rounds of cell division to amplify a population whereas the proventricular epimastigote undergoes a single asymmetric division and is not a self-sustaining population. In each case, the internal arrangement of the nucleus and kinetoplast is shown for 1K1N and 2K2N cells before cytokinesis. There has been no recent detailed description of dividing salivary gland epimastigotes.

The arrangement of the kinetoplast, nucleus and flagellum in different developmental forms of *T. brucei* is shown in Figure 1. Cells are shown with a single kinetoplast and nucleus (1K1N) and also just before cytokinesis after kinetoplast duplication and mitosis (2K2N) where such forms occur. The two types of change readily apparent are alterations in cell dimensions in the AP and radial axis, and a change in the relative positions of the nucleus and kinetoplast along the AP axis.

**Figure 2:**
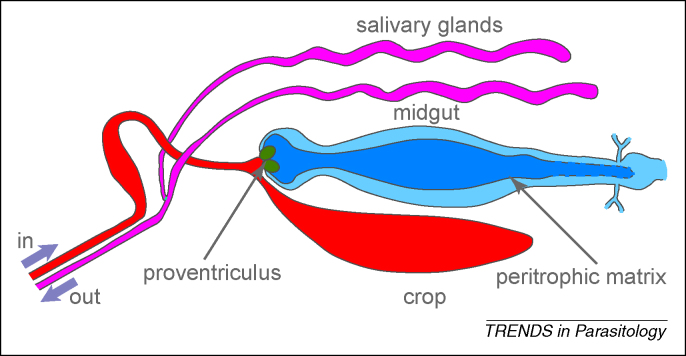
Tsetse viscera. Cartoon of some of the internal organs of a tsetse fly based on a drawing at www.tulane.edu/∼wiser/protozoology/notes/vector.html. Proliferative procyclic forms inhabit the midgut, initially in the endoperitrophic space but rapidly moving into the ectoperitrophic space. From here, there is an anterior movement to the proventriculus, where differentiation to epimastigotes occurs. Proliferation resumes when epimastigotes adhere to the epithelium of the salivary glands where differentiation to mammal-infective metacyclics occurs.

In tsetse flies, a bloodmeal is channelled *via* the food canal, first into the crop and then into the lumen of the midgut within the peritrophic matrix, the endoperitrophic space (Figure 2). To survive, trypanosomes in the bloodmeal must differentiate into procyclic trypomastigotes, an event that occurs within 24 h. This differentiation can be accurately recapitulated *in vitro,* and occurs over 8–10 h in a G1 cell-cycle arrest; this is by far the best-characterised differentiation in trypanosomes [22]. In terms of cellular morphology, the starting and final cells are trypomastigotes, but the position of the kinetoplast along the AP axis is different; in the bloodstream form, it is close to the posterior pole, whereas it is approximately halfway between the nucleus and posterior pole in the procyclic form. Changes in morphology have been investigated in detail using *in-vitro* differentiation [34]. The change in position of the kinetoplast results from: (i) selective elongation of the microtubules at the posterior end of the cell during the differentiation process that effectively moves the posterior pole away from the kinetoplast; and (ii) decrease in the distance between the kinetoplast and nucleus [34]. A second consequence of the differentiation is that the pattern of kinetoplasts and nuclei along the AP axis in a 2K2N cell changes from KKNN in the bloodstream form to KNKN in the procyclic form (Figure 1). It is unclear if there is specific directed movement of organelles and, if so, what process affects the movement.

Invasion of the ectoperitrophic space occurs within a few days of infection [35]. Direct evidence for secondary colonisation of the ectoperitrophic space by the midgut trypomastigotes emerged from studies by Gordon using systematic serial sections of the infected fly [36]. However, the exact route taken by trypanosomes from the endo- to ectoperitrophic space remained contentious for some years [37]. Suggestions included a route *via* the posterior end of the peritrophic matrix, which is ruptured by the rectal spines in the hindgut; penetration at the medial section of the midgut; and penetration of the soft anterior peritrophic matrix close to the proventriculus, which is also the route for re-entering the endoperitrophic space for migratory trypanosomes *en route* to the salivary glands. Electron micrographs convincingly demonstrated that *T. brucei* exited the endoperitrophic space by passage through the membrane over the central two-thirds of the anterior midgut in *Glossina morsitans* [37,38]. It is not known whether a viable trypanosome population remains in the lumen, or whether only the ectoperitrophic space population proliferates. The procyclic trypomastigotes in the ectoperitrophic space are significantly longer and thinner than in the midgut lumen [39].

## The proventriculus

The anterior migration of the trypomastigotes within the ectoperitrophic space is followed by the colonisation of the proventriculus by elongated proventricular procyclic trypomastigotes. The length of the cell increases further as it nears the proventriculus [39,40] again coupled with a further reduction in the width of the cell body. Once the cell length has exceeded a value (∼35 μm using *T. b. brucei* J10: Box 2), proliferation ceases and there is a G2 arrest until the cell reaches the proventriculus [39]. Once the procyclic trypomastigotes reach the proventriculus, there is a further elongation and cells reach lengths of up to ∼40 μm (*T. b. brucei* J10) [39] to 60 μm [14] to form mesocyclic trypomastigotes. These cells deviate from the normal sequence of events in the cell cycle in that they have undergone nuclear S-phase, but have not separated their kinetoplasts; this delay in separation may be preparation for building the short new flagellum (see below). It is not known whether the trypanosomes actively migrate through the ectoperitrophic space towards the proventriculus or whether the cells diffuse there as the number increases. There is some evidence that the elongation of the cell body is in response to proximity to the proventriculus because cells removed from this location return to shorter, proliferating forms [40].

What are the alterations in the cytoskeleton during the differentiation to mesocyclic forms? In the proventriculus, the extension in length and decrease in width occurs during a single cell cycle [39]. The increase in length can be explained by elongation of microtubules to extend the cytoskeleton and/or by sliding of microtubules relative to each other. Normal extension of any microtubule occurs predominantly at the plus end and in trypanosomes would extend the posterior end of the cell. The decrease in diameter is not a result of reduced spacing between sub-pellicular microtubules [39]. Therefore, the decrease in diameter must occur *via* removal of microtubules and/or by sliding of microtubules past one another to the extent that the number of overlapping microtubules decreases. The decrease in the number of overlapping microtubules would also result in an increase in cell length. The resolution of how the cytoskeleton alters will provide evidence on whether the cell can remove microtubules from the sub-pellicular array by depolymerisation.

The differentiation of the long trypomastigote to a short epimastigote (the form believed to colonise the salivary gland) involves a reduction in length by approximately two-thirds, but no substantial increase in cell diameter. This transformation is achieved through an asymmetric division that produces one long and one short daughter epimastigote; the long epimastigote daughter appears to die [39,40]. Asymmetrically dividing *T. brucei* cells were identified by Lewis and Langridge while investigating the morphotypes in the foregut and saliva of *G. morsitans* [12]. An earlier study [9] also demonstrated that *T. b. gambiense* could initiate a form of asymmetric cellular division to produce two unequal-sized daughter epimastigotes. The dramatic reduction in cell length is achieved through an asymmetric segregation of microtubules, as opposed to a loss of microtubules from the sub-pellicular array. Neither form of epimastigote proliferates in the proventriculus, so the trypanosome population in the proventriculus is not self-sustaining and requires continual renewal from the procyclic trypomastigotes in the ectoperitrophic space.

How does the reversal of positions of the kinetoplast and nucleus along the AP axis occur during the differentiation of trypomastigotes to epimastigotes? Most cells in the proventriculus are elongated trypomastigotes with oval or elongated nuclei [39]. As these cells increase in length, the distance of the nucleus and kinetoplast from the anterior pole increases, and the nucleus elongates significantly and takes on a ‘sausage’ shape. This change is possibly enforced on the nucleus by the diminishing diameter of the cell which finally adopts a ‘tadpole’ shape with the posterior pole increasing in diameter and the remainder of the cell decreasing in diameter. The transition to epimastigotes occurs at the same time as separation of daughter kinetoplasts and mitosis. Throughout the process, the distance between the kinetoplast and posterior pole of the cell remains relatively constant. As the transition starts, the distance between the nucleus and the posterior of the cell decreases until the nucleus is positioned in the posterior end of the cell, and the nucleus rounds up and divides. By this point, the nucleus is closer to the posterior pole than to the kinetoplasts and, as cytokinesis occurs, two daughter epimastigote cells are produced [39] (Figure 1). The divided kinetoplast remains in close proximity to the anterior–lateral aspect of the nucleus, with one daughter kinetoplast in a juxta-anterior position. At this stage, the nucleus is already clearly dividing and, as the asymmetric division progresses, the posterior-most daughter kinetoplast is located between the divided nuclei. The anterior kinetoplast remains in close proximity to the anterior pole of the divided nucleus, and these go on to be the nucleus and kinetoplast of the post-cytokinesis long epimastigote. The posterior nucleus–kinetoplast pairs along with the new short flagellum to eventually become the short epimastigote (Figure 1). The use of the new flagellum in short epimastigotes raises the possibility that it is constructed to include components necessary for the subsequent attachment to the salivary gland epithelium.

The asymmetric division requires several coordinated changes in cell shape. The narrowing and elongation of the anterior part of the cell occurs without a decrease in the spacing between microtubules, and so must occur through removal of many microtubules or by sliding of microtubules relative to one another. The sliding of microtubules could explain the increase in length. The increase in diameter at the posterior end of the cell must result from the selective nucleation of additional microtubules within the sub-pellicular array at the posterior end. The transition between morphotypes is most readily explained by movement of the nucleus within the cytoplasm to a position posterior to the kinetoplasts; this mechanism does not require drastic remodelling of the sub-pellicular microtubule array. However, a drastic remodelling of the sub-pellicular microtubules in response to movement of the basal bodies and kinetoplast cannot be ruled out.

### How is the asymmetry in the cell division established?

In the cell-division cycle that contains the asymmetric division, the new flagellum emerges from the flagellar pocket after S-phase late in the cell cycle, after the nucleus has migrated to the posterior end of the cell. The new flagellum is short, and the flagella connector progresses only a short distance down the old flagellum. If the flagella connector marks the site of furrow ingression (as it might do in procyclics), then the asymmetric division is achieved by limiting the growth of the new flagellum, possibly by restricting initiation to late in the cell cycle. In turn, the late initiation might be downstream of a late duplication or division of the basal bodies, seen as a late separation of kinetoplasts. The proventricular cells contrast with procyclic- and bloodstream-form trypomastigotes where separation of kinetoplasts occurs during nuclear S-phase.

## Production of infective metacyclics in the salivary glands

The morphotypes in the salivary glands have been divided into four stages: attached epimastigotes, premetacyclics, and nascent metacyclics, as well as mature free metacyclics [17,14] (Figure 1). The attachment occurs through an elaboration of the membrane of the flagellum [20] that forms junctional complexes with salivary gland epithelial cell microvilli [14]. The attached epimastigotes possess an elongated posterior process and are actively dividing. The nucleus is oval-to-round, and the kinetoplast in the 1N1K cell positioned juxta-anterior or juxta-lateral to the nucleus.

The differentiation of epimastigotes to premetacyclic trypomastigotes occurs while the cells remain attached to the epithelium, but is otherwise not well characterised [17,20,41]. It remains uncertain whether the exchange in the positions of the nucleus and kinetoplast on the AP axis occurs as a result of an altered conformation before cytokinesis or in a non-dividing epimastigote. The attached premetacyclics are characterised as nascent metacyclics when they arrest in G1 before release from the epithelium [14,20,39,41]. A detailed description of differentiation processes occurring in the salivary gland poses the greatest technical challenge due to the difficulties not only in obtaining material, but also in the imaging of individual attached cells. The importance of an improved description of events in the salivary glands is twofold; (i) it remains the least-characterised part of the life cycle, and (ii) the salivary glands are the location of genetic exchange [42] and nothing is known about the cell biology of this in trypanosomes.

## Fusing two closed cages

Tsetse flies infected with two different isolates of *T. brucei* can produce metacyclic trypomastigotes that are hybrid progeny [43]. Thus, genetic exchange occurs within the tsetse fly. The pattern of inheritance of heterozygous loci is Mendelian [44,45], providing strong evidence that sexual genetic exchange occurs. The characterisation of this genetic exchange is important not only for an understanding of the spread of traits such as human infectivity within populations, but also to the development of a system for *in-vitro* crosses providing a simpler forward genetics than currently available. Three cellular events are central to genetic exchange: (i) a meiotic cell cycle including homologous recombination; (ii) fusion of two nuclei; and (iii) reconstitution of diploidy. There is an implicit requirement for cell fusion to occur for random assortment of chromosomes and for mitochondria to fuse for genetic exchange between kinetoplasts (mitochondrial DNA) [32,46,47]. Cells undergoing meiosis and/or cell fusion have not been definitively identified, and the temporal order of meiosis and cell fusion is unknown. The location of genetic exchange within the tsetse fly was investigated in a cross using parental lines expressing different coloured fluorescent proteins. Trypanosomes expressing both fluorescent proteins were observed in the salivary glands, but not in the preceding developmental forms [42]. Part of the problem in identifying the cells undergoing meiosis is that genetic exchange is not an obligatory part of the life cycle, and often only parental genotypes are recovered after passage through tsetse flies. Meiotic cells must be distinguished from the background population of mitotically proliferating epimastigotes present in the salivary glands, and this requires specific tools.

Meiosis and regeneration of the diploid in trypanosomes has two problems. First, there is a problem of the unitary organelles, in particular the complex of the kinetoplast, tripartite attachment complex, basal body and flagellum [35]. Any meiotic cell cycle contains S-phase followed by the first division that separates the homologues, and then the second division that separates sister chromatids. Thus, the products of meiosis could be four haploid cells that have to share two flagella, which may lead to aflagellate cells after the second meiotic division. Fusion of diploid cells before meiosis leads to the reverse problem: after meiotic S-phase the cell would have four flagella. The requirement for mitochondrial fusion to account for mixed minicircles and maxicircles adds to the complexity [46].

Second, there must be a molecular mechanism for fusion of the plasma membrane from two cells and, after the fusion, a route to reverse the crosslinking between microtubules in the sub-pellicular corset, which must relax sufficiently to allow mixing of the two nuclei and mitochondria in a common cytoplasm. At the two extremes, cell fusion could occur side-by-side or end-to-end. In the former, the AP axes of the two cells must be identical to conserve the polarity of the microtubule corset. Membrane fusion must be followed by loosening of the inter-microtubule connections so that the two cylinders of microtubules become one. End-to-end joining would probably involve fusion of the posterior poles of two cells because fusion involving an anterior pole would compromise the flagellum. The mixing of cytoplasm after fusion of two posterior poles would require a relaxation of the ties that bring the sub-pellicular microtubules to a point at the posterior pole. However, end-to-end fusion would result in anti-parallel microtubules, so the fusion would have to be reversed or followed by bending and a zipping-up of the two cells along the AP axis to make the microtubules parallel.

So far, the changes in trypanosome morphology as the cells differentiate from one life cycle stage to another and the probable events involved in genetic exchange have been discussed. Many of the changes can be explained by alterations to the microtubule arrays already present through sliding of microtubules relative to one another and/or by the addition of new microtubules within the array. There is no evidence that there is substantial depolymerisation of microtubules necessary for any transition, and the dramatic shortening of cell length that occurs as epimastigotes are formed results from an asymmetric division of a pre-existing array.

## Experimental approaches

The extent of understanding of the molecular mechanisms responsible for a biological process depends on the types of experimental approaches available. Thus, most research on trypanosomes uses bloodstream and procyclic forms that are readily cultured. By contrast, some of the most interesting life-cycle stages present in the proventriculus and salivary glands are transitory and non-proliferative, whereas the dividing epimastigote population in the salivary glands is intimately attached to the epithelium, a condition so far irreproducible in culture for *T. brucei*. Successful culture of epimastigotes is available for *T. congolense* and this has recently been used for some molecular characterization of adherence [48]. Infected tsetse flies do not contain many trypanosomes; a heavily infected fly contains about 1 × 10^6^ trypanosomes, equivalent to 100 μl of cultured procyclics. Furthermore, the populations isolated from the proventriculus and salivary glands contain mixtures of cell types. This combination of features means that most experiments are usually designed to enable data to be collected from individual cells. Biochemical analysis is challenging, but not impossible, as demonstrated by the elegant identification of the major cell-surface protein on epimastigotes in the salivary glands [49] and the changes in cell-surface proteins in procyclic forms [50]. Using single-cell assays, it is important to use a sufficient number of cells for a statistical analysis, and a second difficulty is to obtain the full range of developmental stages from tsetse flies. In a recent study that characterised the asymmetric division of proventricular trypomastigotes [39], >1000 flies were dissected over a six-month period to find the 334 flies with infected proventriculi and salivary glands used in the study. Often, this time-consuming aspect of working with tsetse flies is neither realised nor recognised.

Newly developed tools designed to exploit the genome sequence that work at the single-cell level can provide access to processes occurring within the tsetse. The genome sequence allows the production of reagents specific for an individual protein. The protein can act as a marker for a particular process, for example SCC1, which is part of the sister chromatid cohesion complex, was used as a marker for S and G2 phases of the cell cycle [39]. A gene product can be used to identify cells undergoing a particular process such as a protein expressed only during meiosis, an approach successfully adopted in *Giardia* using the SPO11 endonuclease [51]. In *T. brucei*, a successful example is the characterisation of expression of endocytic markers throughout the life cycle [52].

Two approaches can be used to determine whether and where (cell type and subcellular localisation) a protein is expressed. The first is detection in fixed cells using an antibody, and the second is tagging to make a fluorescent protein fusion that usually renders the protein visible in living cells if the polypeptide is sufficiently abundant. Antibodies are routinely raised against recombinant proteins, and production of the antibodies requires knowledge of only the sequence. Tagging of genes is best undertaken at the endogenous locus so that the fluorescent protein is at the N-terminus because this leaves regulatory sequences in the 3′UTR unaffected. Both approaches have advantages and disadvantages, and careful controls are needed to minimise the risk of artefactual results. It is crucial to demonstrate the specificity of an antibody, routinely this should be done using RNAi knockdown.

Gene function can be addressed by removing the protein through targeted gene deletion or, sometimes, through reducing expression by RNAi. Targeted gene deletion is a powerful technique for genes that are developmentally regulated; the knockout can be made conditional by deleting the gene in a life-cycle stage where it is not expressed, and then cycling the trypanosomes through tsetse flies. Successful RNAi knockdown of a trypanosome gene in a tsetse fly has not been reported, but there has been successful tetracycline-induced transgene expression [53] so there is no reason why an RNAi approach should not work. A set of experiments to investigate cell morphology and sex in trypanosomes is described below as a simple example of the approaches outlined above.

## Mechanisms underlying changes in cell morphology

Cell morphology can be described in detail through morphometry and a series of cells assembled to build a model of a morphological transformation. In the case of the asymmetric division that occurs in the proventriculus, the model provides evidence that the major change in the conversion of trypomastigote to epimastigote is movement of the nucleus (as opposed to the kinetoplast) along the AP axis. However, the model does not provide information on the molecular mechanisms underlying movement of the nucleus or a change in shape. One means of obtaining information about how changes in cell shape are achieved is to visualise newly polymerised microtubules. Previously, this was achieved by immuno-electron microscopy of cytoskeletons using a monoclonal antibody (mAb) that recognises the tyrosinylated form of α-tubulin, which is characteristic of newly assembled microtubules [54]. Such an approach is demanding for trypanosomes isolated from tsetse flies because the small number of trypanosomes leads to technical difficulties; so are there complementary or alternative approaches that are easier to use? Microtubule plus end-binding proteins have been used as fluorescent protein fusions to visualise microtubule dynamics [55], it is possible that the same approach could be used to mark growing ends of microtubules in trypanosomes, thereby allowing visualisation of extending microtubules.

## Characterising sex

The identification of events during meiosis can be addressed using an analogous route. The timing of nuclear fusion can be determined by using parental lines carrying different colour fluorescent proteins localised to the nucleus, so that when the nuclei are yellow the two parental nuclei are present in the same cell. A similar approach using proteins localised to the mitochondrial matrix can be used to observe fusion of mitochondria, or proteins localised to the kinetoplast to visualise mixing of the kDNA.

The genome sequence represents a tremendous resource for identification of genes with a function in genetic exchange. Meiosis and cell fusion can be investigated by identifying orthologues of genes with a restricted function in either process. For example, *SPO11* encodes the endonuclease that initiates homologous recombination in the prophase of meiosis I and is expressed only at that point in the life cycle of all eukaryotes in which it has been characterised, and apparent orthologues are present in the genomes of protozoa [56]. Cell fusion can be approached using the *HAP2* gene (E. Gluenz and K. Gull, unpublished) [57]. HAP2 is necessary for fusion of membranes after adhesion of gametes in plants and *Plasmodium* [57]. *SPO11* and *HAP2* genes are good examples of where clear prediction can be made about the phenotype of null mutants—both should be unable to cross—and where the expression will provide insights into the biology, SPO11 expression would identify cells undergoing meiosis and HAP2 expression would identify one of the fusogenic cell types.

## Concluding remarks

Many aspects of trypanosome biology take place in the darker recesses of tsetse flies and have yet to be recapitulated in culture. The small number of trypanosomes in any tsetse and the heterogeneity of cell types present in populations harvested from a single tissue means it is difficult to collect data other than by observations in individual cells. One outcome of the genome project is a greater ease of making tools specific for individual gene products. There is now a prospect of being able to address both the questions outlined above, as well as many others. At the moment, we cannot fully exploit the tremendous resource that is the genome because we are capable of easy interrogation of only a small proportion of the cell types occurring during the life cycle. To understand this pathogen more completely, we need to study all the stages. This may require a corporate investment of many researchers. The move to study pleomorphic bloodstream forms has been a very welcome development, ensuring slender- and stumpy-form analysis. The development of systems to study the stages of the salivary gland is no less necessary: this is, after all, the route out of the darkness and into the host!

## Disclosure statement

The authors have no conflicts of interest.

## Box 1 Trypanosomatid cellular morphologies

The flagellum is at the anterior pole of the cell, and the morphology is defined by the relative positions of the nucleus, the kinetoplast (mitochondrial DNA) (which is physically linked to the basal body) and point of emergence of the flagellum from the cell body. *T. brucei* is restricted to the trypomastigote and epimastigote stages in the red box. Other species of kinetoplastids are characterised by other combinations of morphologies; for example, *T. cruzi* has trypomastigote, epimastigote and amastigote stages, *Leishmania* spp. have promastigote and amastigote forms.

## Box 2 Absolute or relative size?

Very few numbers for the absolute size of cells have been given in this review. This lack of numbers has been used to avoid confusion. Absolute cell lengths and diameters vary with strain and growth conditions. The size of a trypanosome varies with the environment, for example *T. brucei* Lister 427 procyclic forms proliferate at similar rates in the two culture media SDM-79 and DTM, but cell size is different. In *T. brucei* Lister 427, procyclic trypomastigotes are longer than bloodstream trypomastigotes, but the opposite is true in *T. brucei* J10. Proventricular trypomastigotes up to 60 μm were reported for *T. brucei* [14], but none greater that 42 μm were seen in hundreds of infections with *T. brucei* J10 [39].
